# A multicenter, prospective, observational study to determine association of mesangial C1q deposition with renal outcomes in IgA nephropathy

**DOI:** 10.1038/s41598-021-84715-7

**Published:** 2021-03-09

**Authors:** Li Tan, Yi Tang, Gaiqin Pei, Zhengxia Zhong, Jiaxing Tan, Ling Zhou, Dongmei Wen, David Sheikh-Hamad, Wei Qin

**Affiliations:** 1 Division of Nephrology, Department of Medicine, West China Hospital, Sichuan University, Chengdu, Sichuan China; 2 Division of Nephrology, Department of Medicine, Affiliated Hospital of Zunyi Medical University, Medical University, Zunyi, Guizhou, China; 3 Division of Nephrology, Zigong Third People’s Hospital, Zigong, Sichuan China; 4 Division of Nephrology, People’s Hospital of Jianyang, Chengdu, Sichuan China; 5 Section of Nephrology, Department of Medicine, Baylor College of Medicine, Houston, TX USA; 6 West China School of Medicine, Sichuan University, Chengdu, Sichuan China

**Keywords:** Nephrology, Kidney diseases, Glomerular diseases, IgA nephropathy

## Abstract

It was reported that histopathologic lesions are risk factors for the progression of IgA Nephropathy (IgAN). The aim of this study was to investigate the relationships between mesangial deposition of C1q and renal outcomes in IgAN. 1071 patients with primary IgAN diagnosed by renal biopsy were enrolled in multiple study centers form January 2013 to January 2017. Patients were divided into two groups: C1q-positive and C1q-negative. Using a 1: 4 propensity score matching (PSM) method identifying age, gender, and treatment modality to minimize confounding factors, 580 matched (out of 926) C1q-negative patients were compared with 145 C1q-positive patients to evaluate severity of baseline clinicopathological features and renal outcome. Kaplan–Meier and Cox proportional hazards analyses were performed to determine whether mesangial C1q deposition is associated with renal outcomes in IgAN. During the follow-up period (41.89 ± 22.85 months), 54 (9.31%) patients in the C1q negative group and 23 (15.86%) patients in C1q positive group reached the endpoint (50% decline of eGFR and/or ESRD or death) respectively (p = 0.01) in the matched cohort. Significantly more patients in C1q negative group achieved complete or partial remission during the follow up period (P = 0.003) both before and after PSM. Three, 5 and 7-year renal survival rates in C1q-positive patients were significantly lower than C1q-negative patients in either unmatched cohort or matched cohort (all p < 0.05). Furthermore, multivariate Cox regression analysis showed that independent risk factors influencing renal survival included Scr, urinary protein, T1-T2 lesion and C1q deposition. Mesangial C1q deposition is a predictor of poor renal survival in IgA nephropathy.

Trial registration TCTR, TCTR20140515001. Registered May 15, 2014, http://www.clinicaltrials.in.th/index.php?tp=regtrials&menu=trialsearch&smenu=fulltext&task=search&task2=view1&id=1074.

## Introduction

Immunoglobulin A nephropathy (IgAN) is one of the most common glomerulonephritis (GN) worldwide, 20–40% of the patients progress to end stage renal disease (ESRD) within 10–20 years from the onset of the disease^1,2^. The diagnosis of IgAN is based on the preponderance of IgA deposits in the mesangium^3,4^. Low intensity C1q deposition may also be found^2,5–8^. Activation of the complement system locally via the classical, alternate and lectin pathways in glomeruli augments the inflammatory cascade and potentiates tissue injury in IgAN^9,10^. It was reported that mesangial C4d deposition, which is a marker of activation of the lectin pathway, is associated with progression to ESRD in IgAN patients^11^. However, whether the presence of C1q deposition, a critical protein in the classical complement pathway, is associated with IgAN remains largely unknown. Therefore, a multicenter, prospective, observational trial was performed to evaluate the relationships between mesangial C1q deposition and clinicopathological features as well as renal outcomes in IgAN patients.

## Results

### Demographic and clinicopathlogical features of the patients

**Figure 1:**
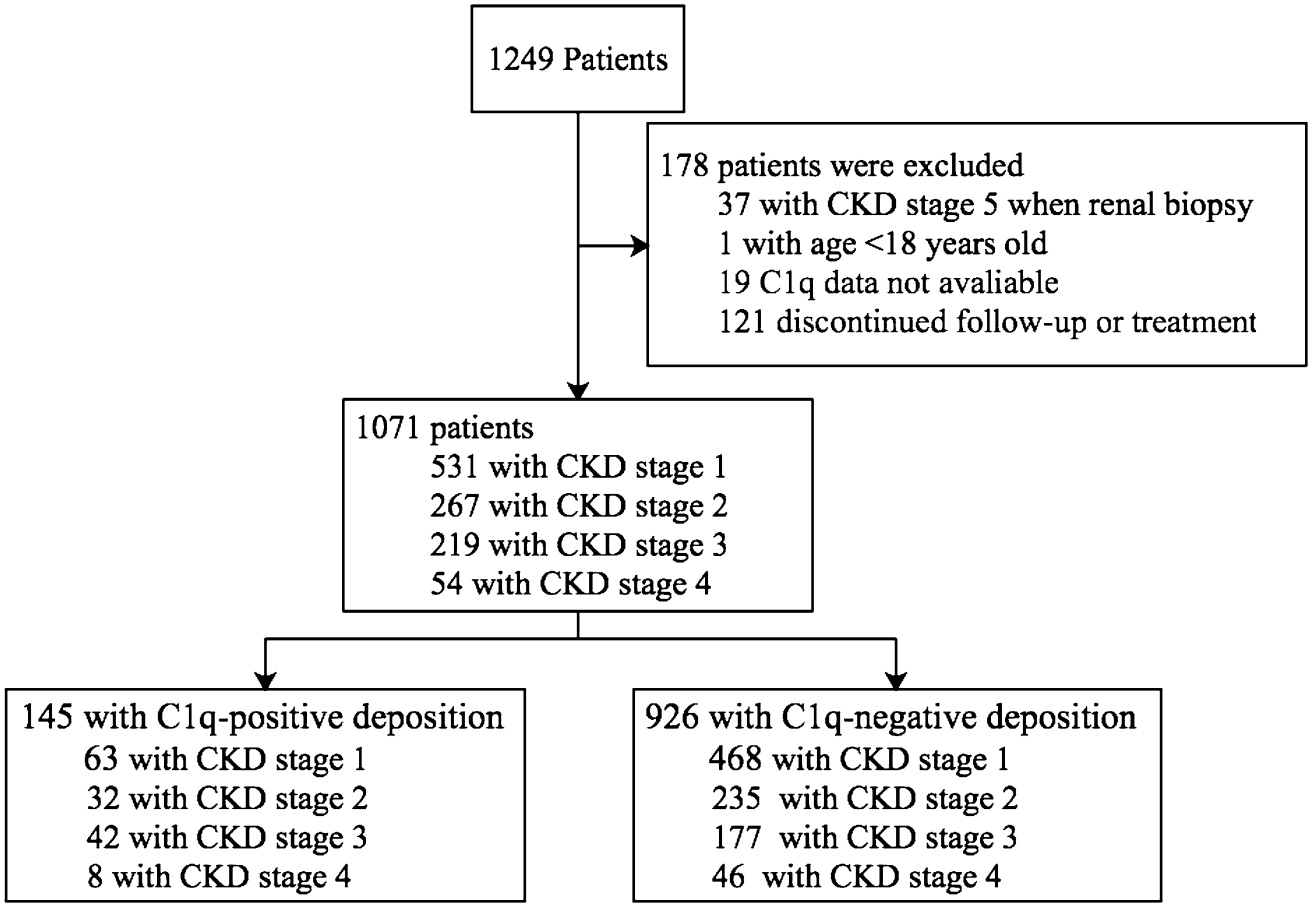
Study profile. *CKD* chronic kidney disease.

1249 IgAN patients from the study centers were enrolled. A total of 178 patients were excluded because of incomplete data, and 1071 patients were included in the analysis (Fig. 1). The average follow-up period was 40.90 ± 24.19 and 41.89 ± 22.85 months before and after adjustment for propensity scores, respectively. Comparison between C1q-positive (n = 145, 13.54%; 1 + , n = 114; 2 + , n = 30; 3 + , n = 1) and C1q-negative (n = 926, 86.46%) patients indicated that C1q deposition was associated with more severe clinical and pathologic features, such as higher level of mean arterial blood pressure (MAP), 24 h urine protein, and lower level of eGFR and serum albumin (Table 1). Corticosteroids were more frequently used in patients with C1q deposition (42.07% vs 35.53%; see Table 1). After 1:4 PS matching, 145 C1q-positive patients were matched with 580 C1q-negative patients. The standardized mean difference was 0.213 before matching, decreased to 0.065 after matching for age, gender and treatment, consistent with corrected bias between the C1q positive and negative patients. Significant differences in eGFR, IgG deposition, IgM deposition, C4 deposition, E1, T1/T2 and C1/C2 lesions were also observed between C1q positive and negative patients (Tables 1 and 2; Supplementary Tables S1 and S2).

**Table 1:** Demographic and clinical features of IgAN patients.

Characteristics	All (n = 1071)	Groups				
		C1q-positive (n = 145)	Unmatched cohort C1q-negative (n = 926)	P value	Matched Cohort (1:4 PSM) C1q-negative (n = 580)	P value
**Clinical**						
Age (year)	33.80 ± 10.96	33.18 ± 10.94	33.89 ± 10.97	0.466	33.99 ± 11.24	0.431
Male (%)	464(43.32)	57(39.31)	407(43.95)	0.290	266 (45.86)	0.156
SBP (mmHg)	130.63 ± 20.27	133.50 ± 21.36	130.18 ± 20.07	0.066	130.17 ± 19.38	0.070
DBP (mmHg)	83.58 ± 14.21	85.78 ± 14.92	83.24 ± 14.07	0.045	83.43 ± 14.21	0.078
MAP (mmHg)	99.26 ± 15.35	101.69 ± 16.45	98.88 ± 15.14	0.041	99.01 ± 15.04	0.060
Hypertension (%)	374 (34.92)	58 (40.00)	316 (34.13)	0.168	198 (34.14)	0.186
Serum creatinine (µmol/L)	100.76 ± 53.06	107.60 ± 53.70	99.69 ± 52.90	0.095	99.88 ± 53.49	0.121
eGFR (ml/min per 1.73 m^2^)	87.48 ± 49.72	81.03 ± 34.84	88.49 ± 51.60	0.027	87.26 ± 32.87	0.044
Urinary Protein (g/24 h)	2.67 ± 2.99	3.27 ± 2.74	2.58 ± 3.02	0.010	2.78 ± 3.22	0.092
Serum albumin (g/L)	38.15 ± 12.51	35.90 ± 7.96	38.50 ± 13.05	0.020	38.22 ± 15.62	0.084
Uric acid (µmol/L)	378.79 ± 158.08	377.05 ± 106.72	379.06 ± 164.72	0.887	380.11 ± 190.48	0.852
**CKD stage**				0.049	380.11 ± 190.48	0.059
Stage 1	531 (49.58)	63 (43.45)	468 (50.54)		294 (50.69)	
Stage 2	267 (24.93)	32 (22.07)	235 (25.38)		148 (25.52)	
Stage 3	219 (20.45)	42 (28.97)	177 (19.11)		110 (18.97)	
Stage 4	54 (5.04)	8 (5.52)	46 (4.97)		28 (4.83)	
**Treatment**				0.043		0.951
SC	411 (38.38)	42 (28.97)	369 (39.85)		168 (28.97)	
CS	390 (36.41)	61 (42.07)	329 (35.53)		251 (43.28)	
IT	270 (25.21)	42 (28.97)	228 (24.62)		161 (27.76)	

Values for categorical variables are given as number (percentage); values for continuous variables are given as mean ± standard deviation or median (interquartile range).

*SBP* systolic blood pressure, *DBP* diastolic blood pressure, *MAP* mean arterial pressure, *eGFR* estimated glomerular filtration rate, *CKD* chronic kidney disease, *SC* supportive care group, *CS* corticosteroids, *IT* immunosuppressive therapy.

**Table 2:** Pathologic features of IgAN patients.

Characteristics	All (n = 1071)	Groups				
		C1q-positive (n = 145)	Unmatched cohort C1q-negative (n = 926)	P value	Matched Cohort (1:4 PSM) C1q-negative (n = 580)	P value
**Pathologic**						
**Oxford classification**						
M1	805 (75.16)	118 (81.38)	687 (74.19)	0.062	441 (76.03)	0.171
E1	56 (5.23)	19 (13.10)	37 (4.00)	< 0.001	28 (4.83)	< 0.001
S	542 (50.61)	66 (45.52)	476 (51.40)	0.187	307 (52.93)	0.110
T0	836 (78.06)	102 (70.34)	734 (79.27)	0.007	456 (78.62)	0.044
T1	190 (17.74)	39 (26.90)	151(16.31)		103 (17.76)	
T2	45 (4.20)	4 (2.76)	41 (4.43)		21 (3.62)	
C0	762 (71.15)	92 (63.45)	670 (72.35)	< 0.001	407 (70.17)	0.004
C1	243 (22.69)	33 (22.76)	210 (22.68)		140 (24.14)	
C2	66 (6.16)	20 (13.79)	46 (4.97)		33 (5.69)	
**IgA deposition**				0.530		0.440
1 +	116 (10.83)	20 (13.79)	96 (10.37)		58 (10.00)	
2 +	495 (46.22)	62 (42.76)	433 (46.76)		276 (47.59)	
3 +	454 (42.39)	62 (42.76)	392 (42.33)		244 (42.07)	
4 +	6 (0.56%)	1 (0.69)	5 (0.54)		2 (0.34)	
IgG deposition	97 (9.06)	32 (22.07)	65 (7.02)	< 0.001	40 (6.90)	< 0.001
IgM deposition	521 (48.65)	127 (87.59)	394 (42.55)	< 0.001	251 (43.28)	< 0.001
C3 deposition	876 (81.79)	124 (85.52)	752 (81.21)	0.211	458 (78.97)	0.076
C4 deposition	52 (4.86)	33 (22.76)	19 (2.05)	< 0.001	11 (1.90)	< 0.001

Values for categorical variables are given as number (percentage); values for continuous variables are given as mean ± standard deviation or median (interquartile range).

*M* mesangial proliferation, *E* endocapillary proliferation, *S* segmental sclerosis, *T* tubular atrophy/interstitial fibrosis, *C* crescents.

### Clinical outcomes in IgAN patients

**Figure 2:**
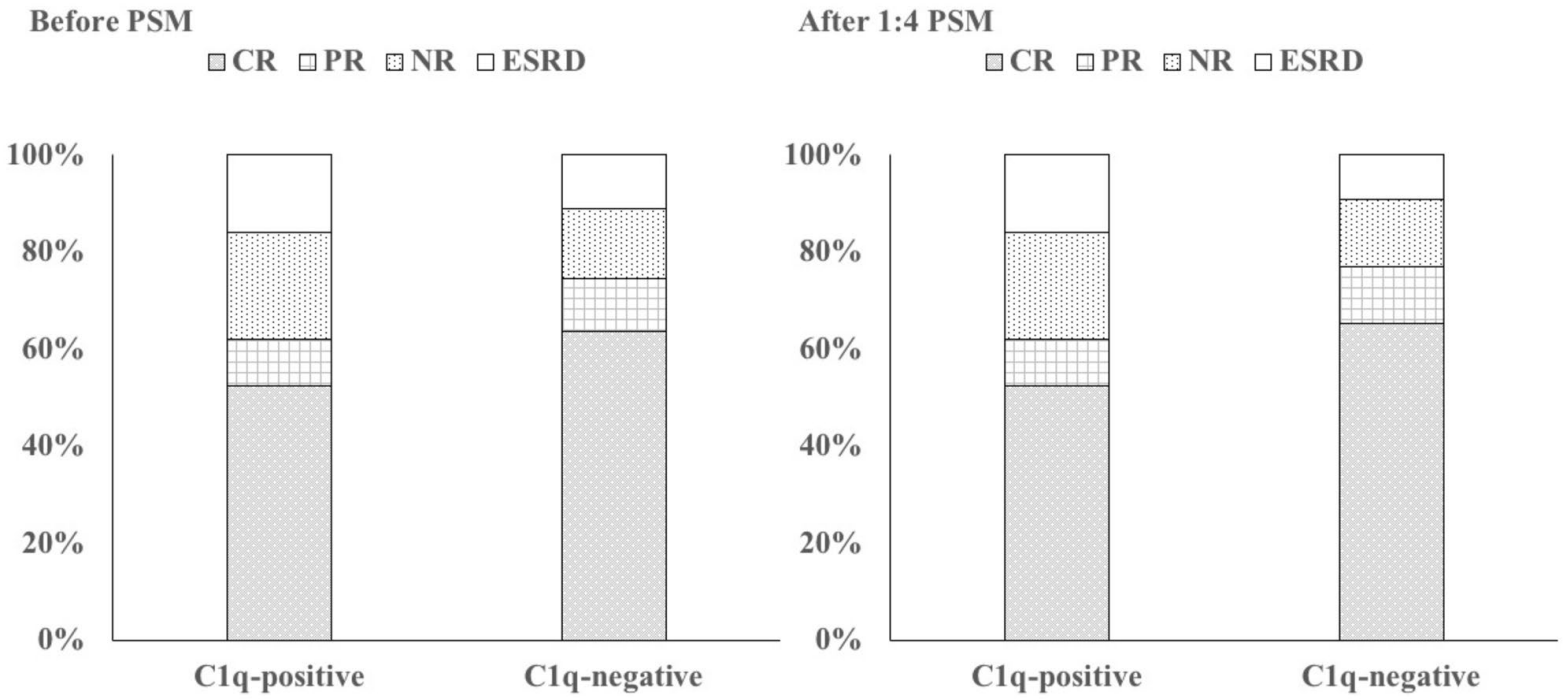
Treatment response and renal outcomes in IgAN patients. *CR* complete remission, *PR* partial remission, *NR* no response, *ESRD* end stage renal disease.

The clinical outcomes in IgAN patients are shown in Table 3; Supplementary Table S3, Fig. 2 and supplementary Fig. S1. Of all the 1071 patients, 62.09% (665) patients achieved CR, 10.83% (116) patients reached PR, 15.41% (165) patients ended in NR, and 11.67% (125) patients progressed to ESRD. Patients in the C1q-positive group had considerably lower incidence of CR and PR and higher incidence of ESRD than is observed in the unmatched C1q negative (p = 0.05) and matched C1q negative (p = 0.003) groups (Table 3 and Supplementary Table S3).

**Table 3:** Clinical outcome of IgAN patients.

	CR	PR	NR	ESRD/death
**Before PS matching (n = 1071)**				
C1-positive (n = 145)	76 (52.4%)	14 (9.7%)	32 (22.1%)	23 (15.9%)
C1-negative (n = 926)	589 (63.6%)	102 (11.0%)	133 (14.4%)	102 (11.0%)
**After PS matching (n = 725, 1:4PSM)**				
C1-positive (n = 145)	76 (52.4%)	14 (9.7%)	32 (22.1%)	23 (15.9%)
C1-negative (n = 580)	378 (65.2%)	69 (11.9%)	79 (13.6%)	54 (9.3%)

Values for categorical variables are given as number (percentage).

*CR* complete remission, *PR* partial remission, *NR* no response, *ESRD* end stage renal disease.

### Patient and renal survival

**Figure 3:**
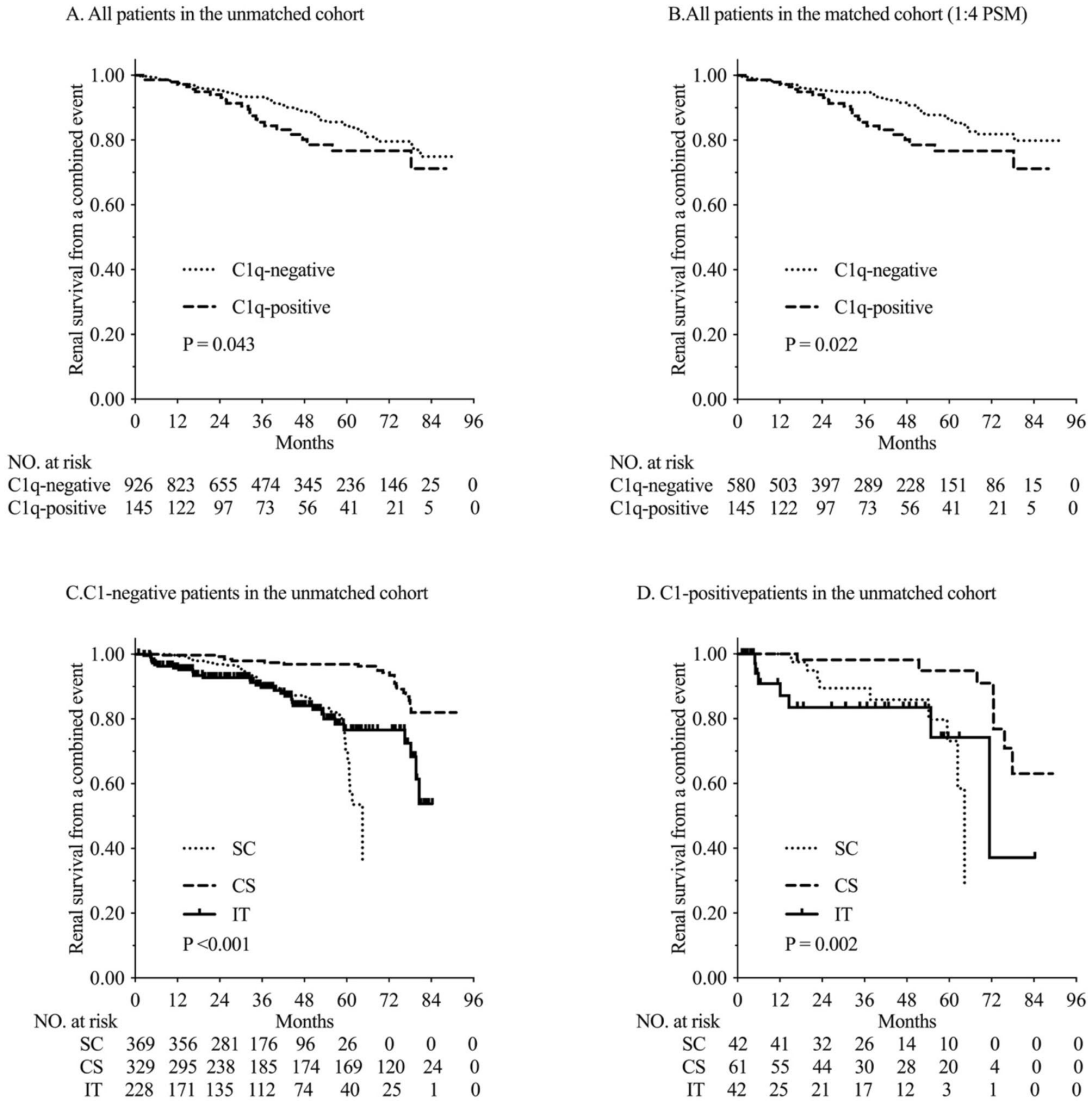
Kaplan–Meier analysis for the probability of composite endpoints.

One patient with C1q deposition died from pneumonia after 2 month of corticosteroids treatment. No patient without C1q deposition died during follow up. More patients with C1q deposition (15.86%) reached the combined endpoint (50% decline of eGFR and/or ESRD or death) during follow up period than patients without C1q deposition before (11.01%) and after (9.31%) PS matching (all p < 0.05). The renal survival rates based on a 50% decline of eGFR, ESRD or death were higher in the C1q-negative patients than in the C1q-positive patients, before (p = 0.04) and after PS matching (p = 0.02). Furthermore, the 3, 5 and 7-year renal survival rates, assessed in terms of combined endpoints, were significantly lower in the C1q-positive patients (88.28%, 84.14% and 83.45%, respectively) compared with the unmatched C1q negative (94.82%, 91.79% and 88.98%, respectively) and matched C1q negative (95.52%, 92.07% and 90.69%, respectively) patients (Fig. 3 and Supplementary Fig. S2).

For C1q-positive and C1q-nepative patients before PS matching, we found that CS could both improve the renal survival compared with SC and IT groups (*P* < 0.001; Fig. 3C,D); while there was no significant difference between SC and IT groups. Further analysis revealed that kidney survival rates in C1q-negative patients received corticosteroids (CS group) were remarkably higher than that in C1q-positive patients (93.01% vs 86.89%, *P* < 0.05; Fig. 3C,D).

### Renal failure predictors

With 50% decline in eGFR and/or ESRD as the combined endpoint, multivariate Cox regression analysis of the unmatched C1q-negative and C1q-positive patients suggested that independent risk factors of poor renal survival include Scr, urinary protein level, T1 or T2 lesion, C1q deposition and C3 deposition. After PS matching, Scr, urinary protein level, T1 or T2 lesion, C1q deposition were also recognized as independent risk factors (Table 4 and Supplementary Table S4).

**Table 4:** Cox proportional hazard model for the primary endpoint in IgA nephropathy patients with CKD.

	Unmatched cohort		Matched cohort	
	HR (95% CI)	P	HR (95% CI)	P
Hypertension	1.226 (0.792–1.898)	0.361	4.397 (2.485–7.782)	< 0.001
Serum creatinine	1.007 (1.004–1.009)	< 0.001	1.008 (1.004–1.012)	< 0.001
Urinary protein	1.093 (1.023–1.168)	0.009	1.100 (1.039–1.164)	0.001
**Pathologic**				
**Oxford**				
M1	0.463 (0.298–0.721)	0.001	2.146 (0.833–5.527)	0.114
E1	1.877 (0.883–3.991)	0.102	3.210 (1.277–8.069)	0.013
S	0.921 (0.637–1.331)	0.662	1.209 (0.722–2.025)	0.470
T0	1	–	1	–
T1	4.305 (2.577–7.192)	< 0.001	3.915 (2.010–7.627)	< 0.001
T2	6.543 (3.581–11.955)	< 0.001	9.677 (3.963–23.631)	< 0.001
C1/C2	0.835 (0.540–1.291)	0.417	0.639 (0.366–1.115)	0.115
C1q deposition	1.659 (1.039–2.650)	0.034	1.948 (1.120–3.387)	0.018
C3 deposition	1.965 (1.186–3.305)	0.011	0.960 (0.463–1.988)	0.912
C4 deposition	0.792 (0.335–1.876)	0.597	0.422 (0.155–1.149)	0.091

*HR* hazard ratio, *95% CI* 95% confidence interval, *M* mesangial proliferation, *E* endocapillary proliferation, *S* segmental sclerosis, *T* tubular atrophy/interstitial fibrosis, *C* crescents.

## Discussion

Activation of the complement system is one of the key participants in the injuries related to IgAN. Activation of the classical complement pathway is initiated after binding of C1q to immune complexes or charged molecules. However, because C1q deposits are not essential for the definitive diagnosis of IgAN, little attention has been paid to its clinical significance. Thus, further research is needed to explore the utility of C1q as a diagnostic or prognostic tool in IgAN patients. C1q deposition rates vary in IgAN patients^11–16^, and possible explanations for the discrepancies among studies may be related to differences in race, age, gender ratio, urine protein and methods of analysis.

The presence of substantial C1q deposition raises the possibility of lupus nephritis with conspicuous IgA deposition. Therefore, patients with lupus nephritis were excluded in this study if endothelial tubuloreticular inclusions were observed on electron microscopy and/or the presence of positive antinuclear antibodies, as per the Kidney Disease Improving Global Outcomes (KDIGO) and American College of Rheumatology (ACR) guidelines. On one hand, similar results were seen in our study no matter the 1: 4 propensity score matching (PSM) or 1:1 PSM was used in this study after calculating a propensity score (PS) for each study patient. On the other hand, in the present study, we demonstrate that C1q positive patients had worse clinical features than both matched and unmatched C1q negative patients. Patients with C1q deposition presented with lower baseline eGFR, rather than severer proteinuria, consistent with activation of the classic complement pathway as a critical determinant in the pathogenesis and clinical outcomes in IgAN. Some prior studies suggested no association between C1q deposition and clinical features in IgAN patients^11^, while other studies have reported that mesangial C1q deposition is associated with poor renal outcomes in patients with IgAN^11,15^, and the slope of eGFR (ΔeGFR/Month) decline was steeper in the C1q-positive patients over a 4-year follow-up^11^. Other studies have also suggested that patients with C1q or IgM deposition had heavier proteinuria than patients without C1q deposition^15,16^, and in agreement with our study, it was suggested that mesangial C1q deposition was associated with higher Lee’s glomerular grade of IgAN (i.e. severer pathologic changes)^11^. Collectively, our observations and those of others suggest that mesangial C1q deposition portends poor renal survival in IgAN patients.

To our knowledge, our study is the first to report association between C1q deposition and Oxford classification of renal pathological changes (MESTC) in IgAN. We observe higher rates of endocapillary hypercellularity (E1), tubular atrophy or interstitial fibrosis (T1 or T2), cellular or fibrocellular crescents (C1 or C2), IgG deposition, IgM deposition and C4 deposition in the C1q positive patients compared with the C1q negative patients (p < 0.05), consistent with worse pathological features in C1q-positive patients. Formation of glycan-specific IgG or IgM autoantibodies, which recognize undergalactosylated IgA1 molecules leads to C1q deposition and is crucial for the pathogenesis of IgAN^9,17^. Furthermore, this study indicates that C4 deposits in the absence of C1q are rare in IgAN (2% of biopsies), suggesting that C4 is activated through the classical pathway. Based on these findings, we speculate that mesangial C1q deposition may induce persistent inflammatory response, leading to endothelial proliferation and cellular crescent formation. Absent effective therapy, these pathological lesions transform into chronic renal fibrosis. Therefore, mesangial C1q deposition in IgAN patients should be considered as an indicator of more aggressive disease, and thus warrants treatment with corticosteroids or immunosuppression.

We found that patients in the C1q-negative group achieved more clinical remission (CR and PR), and had less progression to ESRD than C1q-positive patients (p < 0.05). Furthermore, 15.86% C1q-positive patients reached with combined endpoint (50% decline of eGFR and/or ESRD or death) at a median of about 3.5 years, which would equate to 84.14% of the cohort at 22 years. Even patients without C1q staining showed severe disease with a predicted 65.32% of the cohort reaching the combined endpoint at 22 years. Besides, in terms of Kaplan–Meier survival analysis, the 3, 5 and 7-year renal survival rates in C1q-negative patients were significantly better than in the C1q-positive patients (p < 0.05). Furthermore, corticosteroids were more frequently used in patients with C1q deposition and corticosteroids could improve the renal survival of them.

Cumulative evidence suggests an association between pathological changes and clinical prognosis in IgAN. Several studies have already reported that impaired kidney function, sustained hypertension and renal tubular interstitial atrophy (T1/ T2) were independent risk factors for poor prognosis in IgAN^15,18,19^. Consistently, COX multivariate analysis in our study suggested that Scr, urinary protein, T1 or T2 lesion and C1q deposition were independent indicators of worse renal outcome in IgAN patients. However, few studies reported about the significance of C1q deposition in the pathogenesis of IgAN. In addition, our study recruited more patients and the follow-up was longer. Lastly, PS matching method was used in the current study to minimize bias between patients, which improved the accuracy and reliability of our results.

Based on the findings of our study, we speculate that activation of the classical pathway of complement system plays an important role in the pathogenesis of IgAN. IgG/IgM and C1q co-deposition may initiate the classical complement pathway through activation of C2, C4 and C3—to form the membrane attack complex (MAC), which eventually causes renal inflammation and fibrosis. Although activation of the alternative or lectin pathways of complement were thought to be more important in the pathogenesis of IgAN^1,9^, our results suggest that focused investigations on activation of the classical complement pathway may be warranted—to better understand the pathogenesis of IgAN.

Although we have found several important findings, limitations of our study should also be noted. First, because of the observational design of this study, it is difficult to determine whether prospective intervention and treatment will affect the prognosis of patients with C1q deposition. Second, all patients included in this study were from a single ethnicity (Han Chinese) and the levels of serum C3 and C4 weren't shown in the study. Finally, although we performed PS matching analysis to minimize bias between C1q positive and negative patients, we could only include age, gender, and treatment modality in the PS model; therefore, other confounding factors may exist. Hence, the association between C1q deposition and renal outcomes in IgAN patients must be interpreted with caution.

In conclusion, glomerular deposition of C1q is associated with worse clinicopathologic features and renal outcomes in IgAN patients. Mesangial C1q deposition is an independent risk factor for poor renal prognosis in IgAN.

## Methods

### Patients

Biopsy-proven primary IgAN from patients aged 18 to 75 years were recruited from 4 nephrology centers (West China Hospital, Affiliated Hospital of Zunyi Medical University, Zigong Third People’s Hospital and People’s Hospital of Mianzu city) between January 2013 and January 2017. Inclusion criteria were: (1) Biopsy-proven diagnosis of IgAN and no less than 10 glomeruli the biopsy specimen; (2) the patient had no history of kidney transplant surgery. Exclusion criteria were: (1) secondary IgAN due to systemic diseases such as systemic lupus erythematosus, Henoch–Schönlein purpura and liver cirrhosis, etc.; (2) C1q nephropathy; (3) pregnancy; (4) hepatitis B virus carriers and other chronic liver diseases; (5) baseline estimated glomerular filtration rate (eGFR) < 15 ml/min/1.73 m^2^. The levels of autoimmune antibodies (such as ANA, anti-dsDNA, anti-ENA and anti-Smith) in all biopsy-proven IgAN patients enrolled in the study were negative, thus, these patients were not diagnosed with systemic lupus erythematosus (SLE) according to American College of Rheumatology Guidelines.

Patients were followed up for at least 12 months or shorter if they reached endpoint of study. Patients were followed up monthly in study centers. This research complies with the principles of the Helsinki Declaration and was approved by The Ethics Committee of West China Hospital of Sichuan University, Affiliated Hospital of Zunyi Medical University, Zigong Third People’s Hospital and People’s Hospital of Mianzu city. Written informed consent was obtained from all patients before enrollment into the study.

### Measurements and renal pathology data

Clinical indexes (gender, age, medical history, blood pressure) and laboratory data such as serum albumin (Alb), serum creatinine (Scr), uric acid (UA), 24 h urinary protein at the time of biopsy were collected. Estimated GFR (eGFR) was evaluated according to CKD-EPI (CKD Epidemiology Collaboration) formula^20^.

**Figure 4:**
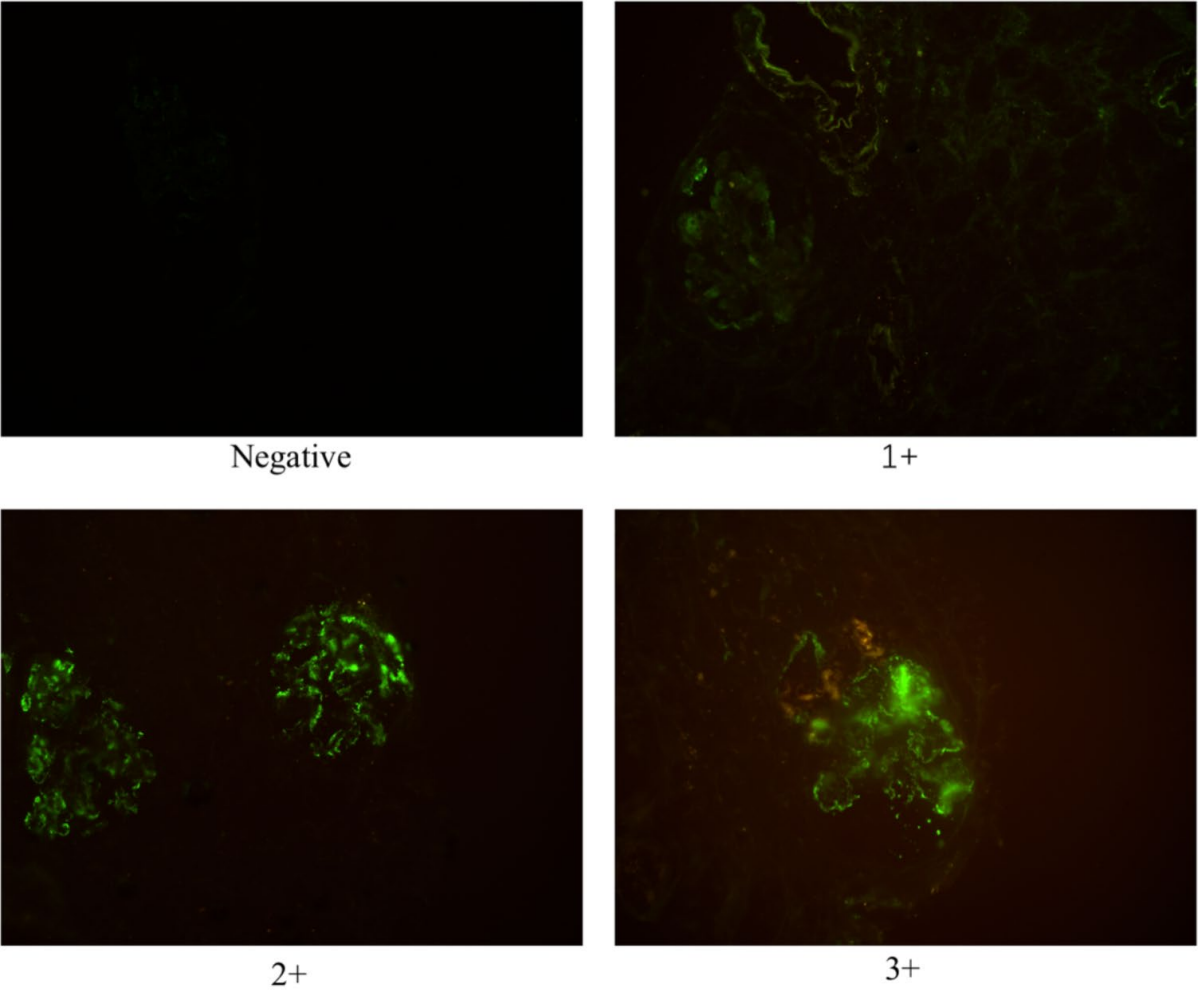
C1q deposition in the mesangium from negative to 3 + (original magnification × 40).

All patients included in the study underwent ultrasound-guided percutaneous renal biopsy. All kidney specimens were obtained by percutaneous needle biopsy and routinely processed for light microscopy, immunofluorescence, and electron microscopy to detect pathologic alterations in the kidney. Serial 2–3 µm thick sections were stained with hematoxylin and eosin (H&E), periodic acid-Schiff, Masson’s trichrome, and periodic acid-methenamine for light microscopy. For immunofluorescence, we used antibodies against IgA, IgG, IgM, C3, C4, C1q, kappa and lambda light chains. The sections were washed thrice with PBS for 5 min each time, incubated with monoclonal rat anti-human C1q antibody (1:100) at 37 °C for 60 min, and washed thrice with PBS for 5 min each time. Then incubated with Alexa Fluor 488 polyclonal goat anti-rat secondary antibody (1:200), and imaged using fluorescence microscope. C1q-positive glomerular deposition was defined as diffuse mesangial and scored as (1 +), (2 +), (3 +); C1q-negative glomerular staining was defined as segmental deposition or absence of staining (shown in Fig. 4).

Renal biopsy specimens of all patients were reviewed by at least two experienced pathologists and two nephrologists. The discrepancy in diagnosis between pathologists and nephrologists was resolved by reviewing the biopsies and coming to a consensus. When there were inconsistencies or doubts among pathologists and clinical investigators, these were submitted to a higher-level pathologist for review. Pathological lesions were graded according to the updated Oxford classification^21^: mesangial hypercellularity (M0/M1); endocapillary hypercellularity (E0/E1); segmental glomerulosclerosis (S0/S1); tubular atrophy / interstitial fibrosis (T0/T1/T2)^21^ and cellular or fibrocellular crescents (C0/C1/C2)^22^.

### Treatment

Treatment modalities were recorded. Patients were treated with 3 different treatment strategies as described previously^19^: supportive care (SC, full dose angiotensin-converting-enzyme inhibitor or angiotensin receptor blockers), supportive care combined with corticosteroids (CS, 0.5–1 mg/kg prednisone daily, tapering down within 6–8 months) and supportive care plus corticosteroids and immunosuppressant therapy (IT, cyclophosphamide 2 mg/kg daily for 3 months, or MMF 1-2 g daily for 6–8 months, or cyclosporine 3–5 mg/kg daily for 6–8 months, or tacrolimus 0.03–0.05 mg/kg daily for 6–8 months).

### Outcomes

Responses to therapy included complete remission (CR), partial remission (PR), no response (NR) and end stage renal disease (ESRD)^19^. CR was defined as urinary protein excretion < 0.5 g/24 h, with < 10% decrease in eGFR relative to baseline. PR was defined as > 50% decrease in proteinuria relative to baseline, with < 10% decrease in eGFR relative to baseline. NR was defined as < 50% decrease in proteinuria relative to baseline, or > 10% decrease in eGFR relative to baseline. ESRD was defined as eGFR < 15 mL/min/1.73 m^2^ or requiring maintenance renal replacement treatment. The primary endpoint was the combined endpoint of a 50% decline of eGFR and/or ESRD or death.

### Statistical analysis

Categorical data were analyzed using Chi-square tests or Fischer’s exact test. Continuous variables were analyzed with unpaired t test or nonparametric Mann–Whitney U test. Categorical variables are presented as frequencies (percentages). Continuous variables are presented as mean ± standard deviation (SD) or median [interquartile range (IQR)]. Multivariate Cox regression analysis was used to evaluate the crude effect of clinical and pathological variables on the renal outcomes before and after adjusting for propensity scores. All statistical analyses were performed using R version 3.3.4 statistical software (The R Foundation for Statistical Computing) and Graphpad Prism7.0. Statistical significance was determined as p < 0.05.

### Propensity score matching

Propensity score matching (PSM) is a statistical matching technique that attempts to estimate the effectiveness of treatments, policies, or other interventions by taking covariates into account^23,24^. PSM reduces the bias due to confounding variables including age, gender, and treatment modality. Therefore, a PSM approach based on the presence of C1q deposition in IgAN patients was used to minimize the effects of confounding factors^24^. The propensity score is calculated by the following conditional probability.

$$ {\text{p (}}x_{i} {)} = {\text{pr (T}} = {1}\left| X \right.{ } = x_{i} {) } = { }\frac{{\exp \left( {r_{0} + r_{1} sex_{i} + r_{2} age_{i} + r_{3} BMI_{i} } \right)}}{{\exp \left( {r_{0} + r_{1} sex_{i} + r_{2} age_{i} + r_{3} BMI_{i} } \right) + 1}} $$

The caliper is defined by the maximum propensity score difference within the matched pair. Three methods of matching individuals with similar propensity scores are presented based on the concept of the caliper in the R package MatchIt^25^. PSM was applied to the KARE data to ensure homogeneity of demographic variables (covariates) between the C1q-positive and C1q-negative groups, using the R package MatchIt.

Since it was necessary to ensure balanced matches between the C1q-positive and C1q-negative groups, we manipulated the caliper (from 0 to 1) by increments of 0.01. We checked the p-values using the paired t-test and the Wilcoxon test to evaluate the homogeneity of the C1q-positive and C1q-negative propensity scores at each caliper increment and for each method of caliper choice. To ensure demographic homogeneity of the C1q-positive and C1q-negative groups, we only considered calipers for which the p-values of both the paired t-test and the Wilcoxon test were larger than 0.05^26^. To ensure balanced matches, a caliper and maximum allowable difference between two groups was defined as 0.1, resulting in a relatively narrow difference between matched subjects^24^. Standardized mean differences were estimated for all baseline covariates before and after matching to assess pre-match imbalance and post-match balance. Although a 1:1 ratio between matched subjects is most commonly used. However, a matching ratio of 1:4 was used for a larger number of control subjects than test subjects in order to improve study power. Finally, after calculating a propensity score (PS) for each study patient, all patients were assigned a 1:4 propensity score matching (PSM) with the occurrence of C1q deposition as the dependent variable to adjust for the observed characteristics of non-randomly assigned patients.

### Ethical approval

The Ethics Committee of West China Hospital of Sichuan University, Affiliated Hospital of Zunyi Medical University, Zigong Third People’s Hospital and People’s Hospital of Mianzu city approved this prospective study. All procedures performed in studies involving human participants were in accordance with the ethical standards of the institutional and/or national research committee and with the 1964 Helsinki declaration and its later amendments or comparable ethical standards.

### Informed consent

Written informed consent was obtained from all patients before enrollment into the study.

## Supplementary Information

Supplementary Figure 1.

Supplementary Figure 2.

Supplementary Table S1.

Supplementary Table S2.

Supplementary Table S3.

Supplementary Table S4.
