# Supplementary material for: A multicenter, prospective, observational study to determine association of mesangial C1q deposition with renal outcomes in IgA nephropathy
Source: Sci Rep. 2021 Mar 9;11:5467. doi: 10.1038/s41598-021-84715-7 (PMC7943768; doi:10.1038/s41598-021-84715-7)
Supplement: Supplementary file 2 — Supplementary Figure 2. [file 41598_2021_84715_MOESM2_ESM.pdf]

A Multicenter, Prospective, Observational Study to Determine Association of Mesangial C1q Deposition with Renal Outcomes in IgA Nephropathy

Li Tan, MD [1,5,6], Yi Tang, MD [1], Gaiqin Pei, MD [1,6], Zhengxia Zhong, MD [2,6], Jiaxing Tan, MD [1,6], Ling Zhou, MD [3, 6], Dongmei Wen, MD [4, 6], David Sheikh-Hamad, MD [5], Wei Qin, MD [1]

[1] Division of Nephrology, Department of Medicine, West China Hospital, Sichuan University, Chengdu, Sichuan, China.

[2] Division of Nephrology, Department of Medicine, Affiliated Hospital of Zunyi Medical University, Medical University, Zunyi, Guizhou, China.

[3] Division of Nephrology, Zigong Third People's Hospital, Zigong, Sichuan, China.

[4] Division of Nephrology, People's Hospital of Jianyang, Chengdu, Sichuan, China.

[5] Section of Nephrology, Department of Medicine, Baylor College of Medicine, Houston, TX, USA.

[6] West China School of Medicine, Sichuan University, Chengdu, Sichuan, China.

Correspondence to: Wei Qin, Division of Nephrology, Department of Medicine, West China Hospital, Sichuan University, Chengdu, Sichuan, China. Tel. 86-28-85422338, Fax +86-028-8542-3341. Email qinweihx@scu.edu.cn.

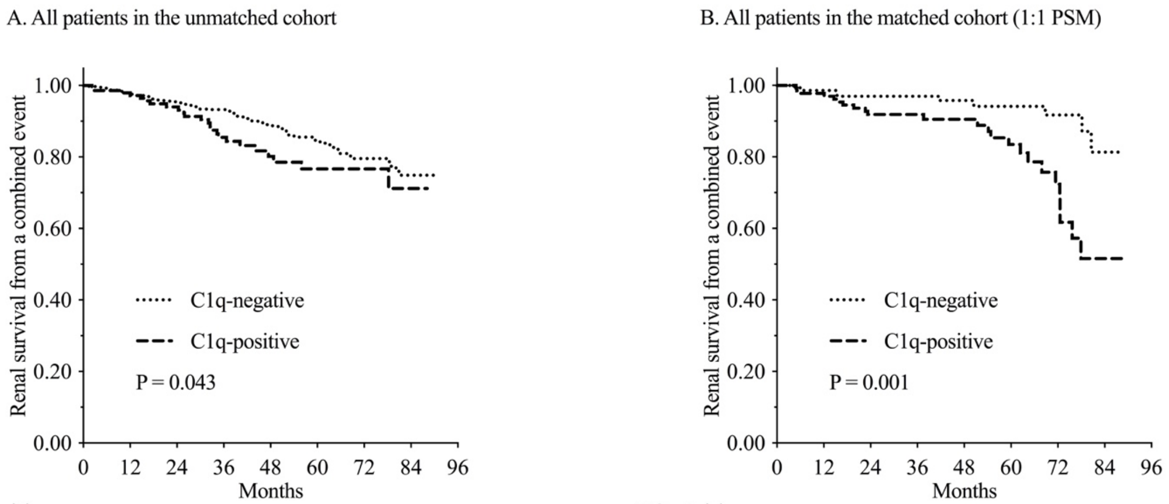

NO. at risk

C1q-negative	926	823	655	474	345	236	146	25	0
C1q-positive	145	122	97	73	56	41	21	5	0

NO. at risk

C1q-negative	145	127	111	86	64	48	30	5	0
C1q-positive	145	122	97	73	56	41	21	5	0

Supplementary Fig. S2. Kaplan-Meier analysis for the probability of composite endpoint.
