# Supplementary material for: A multicenter, prospective, observational study to determine association of mesangial C1q deposition with renal outcomes in IgA nephropathy
Source: Sci Rep. 2021 Mar 9;11:5467. doi: 10.1038/s41598-021-84715-7 (PMC7943768; doi:10.1038/s41598-021-84715-7)
Supplement: Supplementary file 3 — Supplementary Table S1. [file 41598_2021_84715_MOESM3_ESM.pdf]

A Multicenter, Prospective, Observational Study to Determine Association of Mesangial C1q Deposition with Renal Outcomes in IgA Nephropathy

Li Tan, MD [1,5,6], Yi Tang, MD [1], Gaiqin Pei, MD [1,6], Zhengxia Zhong, MD [2,6], Jiaxing Tan, MD [1,6], Ling Zhou, MD [3,6], Dongmei Wen, MD [4,6], David Sheikh-Hamad, MD [5], Wei Qin, MD [1]

[1] Division of Nephrology, Department of Medicine, West China Hospital, Sichuan University, Chengdu, Sichuan, China.

[2] Division of Nephrology, Department of Medicine, Affiliated Hospital of Zunyi Medical University, Medical University, Zunyi, Guizhou, China.

[3] Division of Nephrology, Zigong Third People's Hospital, Zigong, Sichuan, China.

[4] Division of Nephrology, People's Hospital of Jianyang, Chengdu, Sichuan, China.

[5] Section of Nephrology, Department of Medicine, Baylor College of Medicine, Houston, TX, USA.

[6] West China School of Medicine, Sichuan University, Chengdu, Sichuan, China.

Correspondence to: Wei Qin, Division of Nephrology, Department of Medicine, West China Hospital, Sichuan University, Chengdu, Sichuan, China. Tel. 86-28-85422338, Fax +86-028-8542-3341. Email qinweihx@scu.edu.cn.

TableS1. Demographic and Clinical Features of IgAN Patients.

Characteristics	Groups					
	**All (n =1071)**	**C1q-positive (n = 145)**	**Unmatched cohort C1q-negative (n=926)**	**P value**	**Matched Cohort (1:1 PSM) C1q-negative (n=145)**	**P value**
Clinical						
Age (year)	33.80 ± 10.96	33.18 ± 10.94	33.89 ± 10.97	0.466	34.05 ± 11.74	0.409
Male (%)	464（43.32）	57（39.31）	407（43.95）	0.290	57 (39.31)	0.962
SBP (mmHg)	130.63 ± 20.27	133.50 ± 21.36	130.18 ± 20.07	0.066	131.89 ± 21.58	0.167
DBP (mmHg)	83.58 ± 14.21	85.78 ± 14.92	83.24 ± 14.07	0.045	82.65 ± 14.61	0.153
MAP (mmHg)	99.26 ± 15.35	101.69 ± 16.45	98.88 ± 15.14	0.041	99.06 ± 16.08	0.143
Hypertension (%)	374 (34.92)	58 (40.00)	316 (34.13)	0.168	51 (35.17)	0.422
Serum creatinine (μmol/L)	100.76 ± 53.06	107.60 ± 53.70	99.69 ± 52.90	0.095	100.91 ± 54.89	0.296
eGFR (ml/min per 1.73 m^2)	87.48 ± 49.72	81.03 ± 34.84	88.49 ± 51.60	0.027	87.12 ± 34.27	0.043
Urinary protein (g/24h)	2.67 ± 2.99	3.27 ± 2.74	2.58 ± 3.02	0.010	2.58 ± 2.47	0.026
Serum albumin (g/L)	38.15 ± 12.51	35.90 ± 7.96	38.50 ± 13.05	0.020	37.94 ± 6.95	0.021
Uric acid (μmol/L)	378.79 ± 158.08	377.05 ± 106.72	379.06 ± 164.72	0.887	371.92 ± 109.58	0.812
CKD stage				0.049		0.399
Stage 1	531 (49.58)	63 (43.45)	468 (50.54)		68 (46.90)	
Stage 2	267 (24.93)	32 (22.07)	235 (25.38)		40 (27.59)	
Stage 3	219 (20.45)	42 (28.97)	177 (19.11)		31 (21.38)	
Stage 4	54 (5.04)	8 (5.52)	46 (4.97)		6 (4.14)	
Treatment				0.043		0.972
SC	411 (38.38)	42 (28.97)	369 (39.85)		42 (28.97)	
CS	390 (36.41)	61 (42.07)	329 (35.53)		61 (42.07)	
IT	270 (25.21)	42 (28.97)	228 (24.62)		42 (28.97)	

Note: Values for categorical variables are given as number (percentage); values for continuous variables are given as mean ± standard deviation or median (interquartile range). Abbreviations: SBP, systolic blood pressure; DBP, diastolic blood pressure; MAP, mean arterial pressure; eGFR, estimated glomerular filtration rate; CKD, chronic kidney disease; SC, supportive care group; CS, corticosteroids; IT, immunosuppressive therapy.
