# Supplementary material for: A multicenter, prospective, observational study to determine association of mesangial C1q deposition with renal outcomes in IgA nephropathy
Source: Sci Rep. 2021 Mar 9;11:5467. doi: 10.1038/s41598-021-84715-7 (PMC7943768; doi:10.1038/s41598-021-84715-7)
Supplement: Supplementary file 4 — Supplementary Table S2. [file 41598_2021_84715_MOESM4_ESM.pdf]

A Multicenter, Prospective, Observational Study to Determine Association of Mesangial C1q Deposition with Renal Outcomes in IgA Nephropathy

Li Tan, MD [1,5,6], Yi Tang, MD [1], Gaiqin Pei, MD [1,6], Zhengxia Zhong, MD [2,6], Jiaxing Tan, MD [1,6], Ling Zhou, MD [3, 6], Dongmei Wen, MD [4, 6], David Sheikh-Hamad, MD [5], Wei Qin, MD [1]

[1] Division of Nephrology, Department of Medicine, West China Hospital, Sichuan University, Chengdu, Sichuan, China.

[2] Division of Nephrology, Department of Medicine, Affiliated Hospital of Zunyi Medical University, Medical University, Zunyi, Guizhou, China.

[3] Division of Nephrology, Zigong Third People's Hospital, Zigong, Sichuan, China.

[4] Division of Nephrology, People's Hospital of Jianyang, Chengdu, Sichuan, China.

[5] Section of Nephrology, Department of Medicine, Baylor College of Medicine, Houston, TX, USA.

[6] West China School of Medicine, Sichuan University, Chengdu, Sichuan, China.

Correspondence to: Wei Qin, Division of Nephrology, Department of Medicine, West China Hospital, Sichuan University, Chengdu, Sichuan, China. Tel. 86-28-85422338, Fax +86-028-8542-3341. Email qinweihx@scu.edu.cn.

TableS2. Pathologic Features of IgAN Patients.

Characteristics	All (n =1071)	C1q-positive (n = 145)	Unmatched cohort C1q-negative (n=926)	P value	Matched Cohort (1:1 PSM) C1q-negative (n=145)	P value
		Groups				
Pathologic						
Oxford Classification						
M1	805 (75.16)	118 (81.38)	687 (74.19)	0.062	112 (77.24)	0.384
E1	56 (5.23)	19 (13.10)	37 (4.00)	<0.001	8 (5.52)	0.026
S	542 (50.61)	66 (45.52)	476 (51.40)	0.187	79 (54.48)	0.127
T0	836 (78.06)	102 (70.34)	734 (79.27)	0.007	120 (82.76)	0.044
T1	190 (17.74)	39 (26.90)	151(16.31)		23 (15.86)	
T2	45 (4.20)	4 (2.76)	41 (4.43)		2 (1.38)	
C0	762 (71.15)	92 (63.45)	670 (72.35)	<0.001	109 (75.17)	0.044
C1	243 (22.69)	33 (22.76)	210 (22.68)		27 (18.62)	
C2	66 (6.16)	20 (13.79)	46 (4.97)		9 (6.21)	
IgA deposition				0.530		0.942
1+	116 (10.83)	20 (13.79)	96 (10.37)		21 (14.48)	
2+	495 (46.22)	62 (42.76)	433 (46.76)		62 (42.76)	
3+	454 (42.39)	62 (42.76)	392 (42.33)		60 (41.38)	
4+	6 (0.56%)	1 (0.69)	5 (0.54)		2 (1.38)	
IgG deposition	97 (9.06)	32 (22.07)	65 (7.02)	<0.001	14 (9.66)	0.004
IgM deposition	521 (48.65)	127 (87.59)	394 (42.55)	<0.001	72 (49.66)	<0.001
C3 deposition	876 (81.79)	124 (85.52)	752 (81.21)	0.211	119 (82.07)	0.426
C4 deposition	52 (4.86)	33 (22.76)	19 (2.05)	<0.001	8 (5.52)	<0.001

Note: Values for categorical variables are given as number (percentage); values for continuous variables are given as mean ± standard deviation or median (interquartile range).

Abbreviations: M, mesangial proliferation; E, endocapillary proliferation; S, segmental sclerosis; T, tubular atrophy/interstitial fibrosis; C, crescents.
