# Supplementary material for: A multicenter, prospective, observational study to determine association of mesangial C1q deposition with renal outcomes in IgA nephropathy
Source: Sci Rep. 2021 Mar 9;11:5467. doi: 10.1038/s41598-021-84715-7 (PMC7943768; doi:10.1038/s41598-021-84715-7)
Supplement: Supplementary file 5 — Supplementary Table S3. [file 41598_2021_84715_MOESM5_ESM.pdf]

A Multicenter, Prospective, Observational Study to Determine Association of Mesangial C1q Deposition with Renal Outcomes in IgA Nephropathy

Li Tan, MD [1,5,6], Yi Tang, MD [1], Gaiqin Pei, MD [1,6], Zhengxia Zhong, MD [2,6], Jiaxing Tan, MD [1,6], Ling Zhou, MD [3,6], Dongmei Wen, MD [4,6], David Sheikh-Hamad, MD [5], Wei Qin, MD [1]

[1] Division of Nephrology, Department of Medicine, West China Hospital, Sichuan University, Chengdu, Sichuan, China.

[2] Division of Nephrology, Department of Medicine, Affiliated Hospital of Zunyi Medical University, Medical University, Zunyi, Guizhou, China.

[3] Division of Nephrology, Zigong Third People's Hospital, Zigong, Sichuan, China.

[4] Division of Nephrology, People's Hospital of Jianyang, Chengdu, Sichuan, China.

[5] Section of Nephrology, Department of Medicine, Baylor College of Medicine, Houston, TX, USA.

[6] West China School of Medicine, Sichuan University, Chengdu, Sichuan, China.

Correspondence to: Wei Qin, Division of Nephrology, Department of Medicine, West China Hospital, Sichuan University, Chengdu, Sichuan, China. Tel. 86-28-85422338, Fax +86-028-8542-3341. Email qinweihx@scu.edu.cn.

TableS3. Clinical outcome of IgAN patients.

	CR	PR	NR	ESRD/ Death
Before PS matching (n=1071)				
C1-positive (n=145)	76 (52.4%)	14 (9.7%)	32 (22.1%)	23 (15.9%)
C1-negative (n=926)	589 (63.6%)	102 (11.0%)	133 (14.4%)	102 (11.0%)
After 1:1 PS matching (n=290)				
C1-positive (n=145)	76 (52.4%)	14 (9.7%)	32 (22.1%)	23 (15.9%)
C1-negative (n=145)	101 (69.7%)	18(12.4%)	17 (11.7%)	9 (6.2%)

Note: Values for categorical variables are given as number (percentage). Abbreviations: CR, complete remission; PR, partial remission; NR, no response; ESRD, end stage renal disease.
