# Supplementary material for: A multicenter, prospective, observational study to determine association of mesangial C1q deposition with renal outcomes in IgA nephropathy
Source: Sci Rep. 2021 Mar 9;11:5467. doi: 10.1038/s41598-021-84715-7 (PMC7943768; doi:10.1038/s41598-021-84715-7)
Supplement: Supplementary file 6 — Supplementary Table S4. [file 41598_2021_84715_MOESM6_ESM.pdf]

A Multicenter, Prospective, Observational Study to Determine Association of Mesangial C1q Deposition with Renal Outcomes in IgA Nephropathy

Li Tan, MD [1,5,6], Yi Tang, MD [1], Gaiqin Pei, MD [1,6], Zhengxia Zhong, MD [2,6], Jiaxing Tan, MD [1,6], Ling Zhou, MD [3, 6], Dongmei Wen, MD [4, 6], David Sheikh-Hamad, MD [5], Wei Qin, MD [1]

[1] Division of Nephrology, Department of Medicine, West China Hospital, Sichuan University, Chengdu, Sichuan, China.

[2] Division of Nephrology, Department of Medicine, Affiliated Hospital of Zunyi Medical University, Medical University, Zunyi, Guizhou, China.

[3] Division of Nephrology, Zigong Third People's Hospital, Zigong, Sichuan, China.

[4] Division of Nephrology, People's Hospital of Jianyang, Chengdu, Sichuan, China.

[5] Section of Nephrology, Department of Medicine, Baylor College of Medicine, Houston, TX, USA.

[6] West China School of Medicine, Sichuan University, Chengdu, Sichuan, China.

Correspondence to: Wei Qin, Division of Nephrology, Department of Medicine, West China Hospital, Sichuan University, Chengdu, Sichuan, China. Tel. 86-28-85422338, Fax +86-028-8542-3341. Email qinweihx@scu.edu.cn.

TableS4. Cox Proportional Hazard Model for the Primary Endpoint in IgA Nephropathy Patients with CKD.

	Unmatched cohort		Matched cohort (1:1 PSM)	
	HR (95%CI)	P	HR (95%CI)	P
Hypertension	1.226 (0.792-1.898)	0.361	4.116 (2.390-7.089)	＜0.001
Serum creatinine	1.007 (1.004-1.009)	＜0.001	1.010 (1.006-1.017)	＜0.001
Urinary protein	1.093 (1.023-1.168)	0.009	1.078 (1.020-1.140)	0.008
Pathologic				
Oxford				
M1	0.463 (0.298-0.721)	0.001	2.172 (0.870-5.550)	0.112
E1	1.877 (0.883-3.991)	0.102	2.708 (1.105-6.635)	0.029
S	0.921 (0.637-1.331)	0.662	1.077 (0.65-1.784)	0.774
T0	1	-	1	-
T1	4.305 (2.577-7.192)	＜0.001	3.382 (1.767-6.472)	＜0.001
T2	6.543 (3.581-11.955)	＜0.001	10.901 (4.767-24.931)	＜0.001
C1/C2	0.835 (0.540-1.291)	0.417	0.615 (0.354-1.067)	0.084
C1q deposition	1.659 (1.039-2.650)	0.034	2.106 (1.216-3.647)	0.008
C3 deposition	1.965 (1.186-3.305)	0.011	0.838 (0.406-1.728)	0.632
C4 deposition	0.792 (0.335-1.876)	0.597	0.480 (0.179-1.288)	0.145

Abbreviations: HR, hazard ratio; 95 % CI, 95 % confidence interval; M, mesangial proliferation; E, endocapillary proliferation; S, segmental sclerosis; T, tubular atrophy/interstitial fibrosis; C, crescents.
